# Nanomedicines targeting activation of STING to reshape tumor immune microenvironment and enhance immunotherapeutic efficacy

**DOI:** 10.3389/fonc.2022.1093240

**Published:** 2023-01-18

**Authors:** Shanshan Chen, Anghui Peng, Muhe Chen, Meixiao Zhan

**Affiliations:** ^1^Zhuhai Interventional Medical Center, Zhuhai Precision Medical Center, Zhuhai People’s Hospital, Zhuhai Hospital Affiliated with Jinan University, Jinan University, Zhuhai, China; ^2^Guangdong Provincial Key Laboratory of Tumor Interventional Diagnosis and Treatment, Zhuhai Institute of Translational Medicine, Zhuhai People’s Hospital Affiliated with Jinan University, Jinan University, Zhuhai, China

**Keywords:** nanomedicines, tumor immune microenvironment (TIME), STING agonists, antitumor therapy, innate immune

## Abstract

Immunotherapy has greatly enhanced the effectiveness of cancer treatments, but the efficacy of many current immunotherapies is still limited by the tumor-suppressive immune microenvironment. Multiple studies have shown that activating the stimulation of IFN genes (STING) pathway and inducing innate immunity can significantly impact the tumor immune microenvironment and improve antitumor therapy. While natural or synthetic STING agonists have been identified or developed for preclinical and clinical use, small molecule agonists have limited utility due to degradation and lack of targeting. As such, the delivery and release of STING agonists into tumor tissue is a major challenge that must be addressed in order to further advance the use of STING agonists. To address this challenge, various nanomedicines have been developed. In this paper, we concisely review the antitumor immunotherapeutic mechanisms of STING agonists, highlighting the latest developments in STING agonists and the current progress of nanomedicines for activating STING. We classify the different nanomedicines according to the STING agonists they utilize in order to facilitate understanding of recent advances in this field. Finally, we also discuss the prospects and challenges of this field.

## Introduction

Conventional oncology therapies (such as surgery, chemotherapy, or radiotherapy) often treat tumors at the expense of the patient’s immunity and quality of life. This means that conventional oncology therapies can be harmful to the patient’s body while achieving their therapeutic goals. In the past decade, the field of cancer treatment has undergone an immunological revolution. Immunocellular therapy with lower side effects is receiving increasing attention from researchers as a powerful complement to traditional therapies. Immune checkpoint blockade (ICB) is considered to be the first generation of immunotherapy. The main therapeutic mechanism of ICB is to block the mutual recognition process of surface inhibitory receptors and ligands between tumor cells and T cells. This will enable T cells to exert their cytotoxic effects normally (1). There have been a number of cases showing a clear clinical benefit of ICB (2). However, clinical results show that many patients do not respond to ICB therapy. For example, programmed death-ligand 1 (PD-L1) inhibitors are only 40% to 50% effective in PD-L1 positive metastatic melanoma or lung cancer. Furthermore, despite PD-L1 positivity rates between 40% and 50% in patients with colorectal cancer, programmed death-1(PD-1) inhibitors or PD-L1 inhibitors are still not effective enough for patients (3). The poor response to ICB therapy may be due to the complex microenvironment of tumors, in addition to differences in individual genetic mutations.

The tumor microenvironment (TME) is the complex tissue environment surrounding the tumor cells. It consists of tumor cells, immune cells, stromal cells, vascular cells, other cellular and non-cellular components (4). Depending on the level of pro-inflammatory cytokines and T-cell infiltration in the TME, tumors can be simply classified as “cold” or “hot” tumors (5). Cold tumors have only low levels of T-cell infiltration, resulting in low responsiveness to various immunotherapies (6). Studies have also shown that hot tumors have a higher response rate to immunotherapy (7). Therefore, converting non-inflammatory cold tumors into hot tumors is the key to fully exploit the antitumor effects of immunotherapy. More and more studies suggests that the immune status of these “cold” tumors can be reversed by activating the innate immune system (8).

There is growing evidence that activating one of the innate signaling pathways, the stimulation of IFN genes (STING) signaling pathway, is a promising approach to cancer immunotherapy (9). This approach focuses on the use of STING agonists to activate STING and induce type I interferon (IFN-I) production, ultimately generating a pro-inflammatory response to enhance the antitumor immune response in the TME (10). IFN-I signaling molecules has been shown to enhance the ability of DC cells to activate T cells in a variety of ways, which is critical for the generation of tumor-specific immunity (11). In an experiment led by researchers from the University of Chicago, researchers discovered that intratumoral injection of STING agonists activates STING and produces CD8^+^ cells, which may be used to eliminate *in situ* tumors as well as lung metastases through systemic adaptive immunity (12).

It is currently the primary clinical approach to inject STING agonists directly into tumors by intra-tumoral injections since STING is widely distributed in somatic cells. However, it should be noted that intra-tumor injections may not always be feasible in clinical practice due to uneven drug diffusion and possible drug leakage issues. Besides, the activity of STING is mediated through the delivery of STING agonists into the cytosol of the cell (13). This means that it is necessary to deliver STING agonists intracellularly to ensure maximal biological activity (14). Therefore, it is imperative that these barriers are overcome in order for STING agonists to become a practical and affordable option. By developing advanced nanotechnology-based delivery systems, it is possible to improve and eliminate some of the side effects associated with current therapies.

A brief description of the mechanisms of tumor immunotherapy associated with activation of the STING pathway will be presented in this article, as well as a summary of recent advances in nanomedicines aimed at activating STING. There is excellent spatiotemporal controllability of nanomedicines incorporating STING agonists, as well as the ability to control immune activation in response to TME signaling. The results of these studies are of significant value in improving some of the limitations and drawbacks of conventional immunotherapy. In addition, they enable more effective translation and application of cancer immunotherapy.

## Mechanism of antitumor immunotherapy by activation of the cGAS-STING pathway

**Figure 1:**
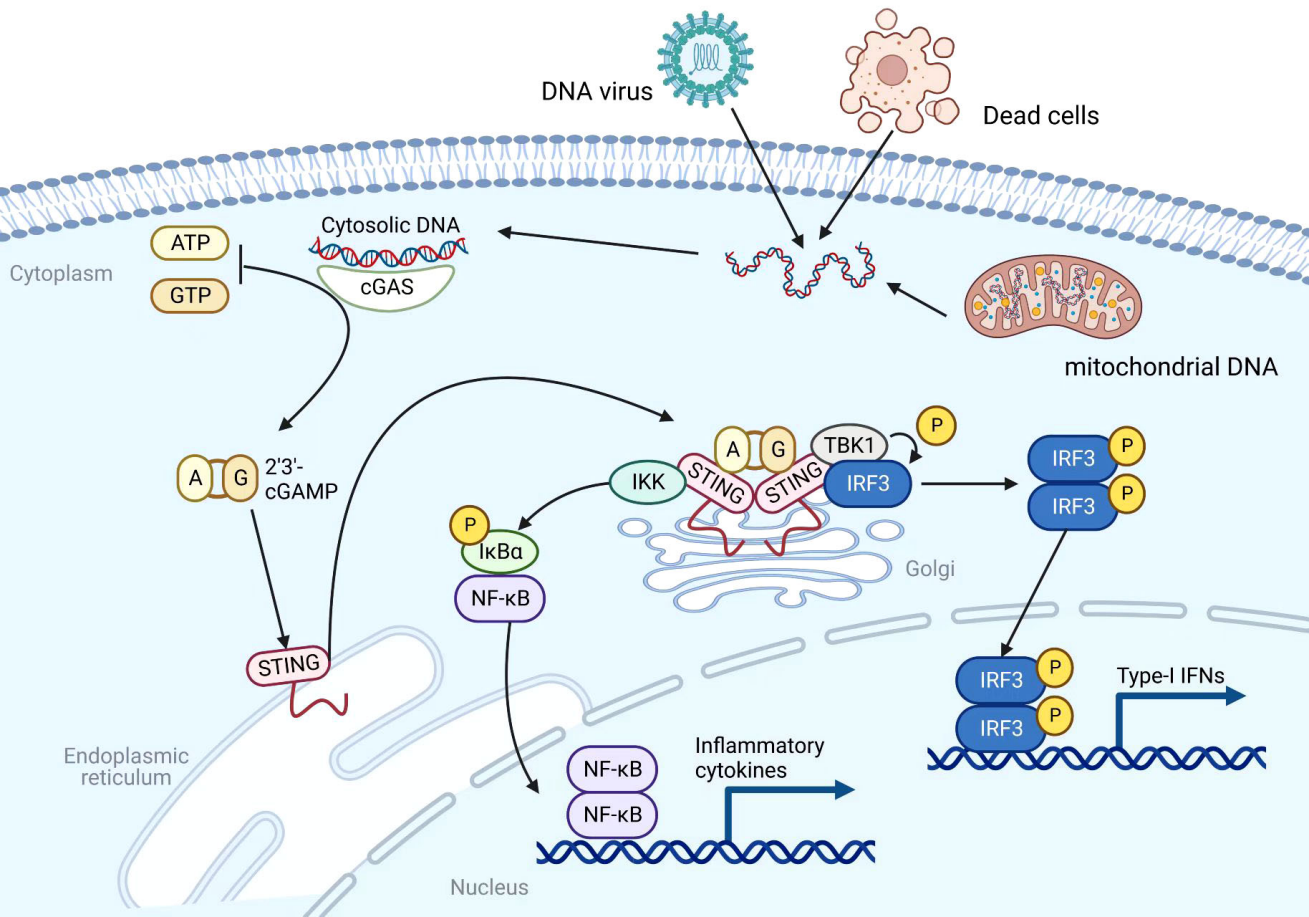
Mechanism of the cGAS-STING pathway. Created with BioRender.com.

Most immunotherapies are aimed at enhancing adaptive antitumor immunity at the present time. It’s important to note that adaptive antitumor immunity is heavily dependent on the strength of the innate immunity (15). Through a number of pattern recognition receptors (PRRs), such as the cytosolic DNA sensor, innate immunity can serve as the first immune barrier of the host against foreign material (16). As a DNA sensor anchored in the endoplasmic reticulum (ER), STING senses DNA leak signals in the cytosol of the cell (17) (**Figure 1**). However, the STING pathway is not actually activated directly by double-stranded DNA (dsDNA). Its activation is predominantly mediated by the second messenger cyclic dinucleotides (CDNs) that is produced by cyclic GMP–AMP synthase (cGAS) (18). As a result of its direct binding to cytosolic dsDNA, cGAS catalyzes the production of cyclic GMP-AMP (cGAMP) in the cell (19, 20). Upon stimulation with cGAMP, the STING molecule changes from monomer to dimer conformation (21). Through the Golgi apparatus, STING dimers are translocated from the ER to the perinuclear microsome. By recruiting and activating TANK-binding kinase 1 (TBK1), STING is able to phosphorylate interferon regulatory transcription factor 3 (IRF3), which further raises the expression of IFN-I (22). In addition, STING could activate the nuclear factor kappa-light-chain-enhancer of activated B cells (NF-κB) pathway by binding to IκB kinase (IKK) and NF-κB-inducing kinase (NIK) (23). Activated NF-κB pathways work together with the TBK1-IRF3 pathway in order to induce IFN-I. There are a number of immune-stimulatory functions that are associated with IFN-I, such as the ability to facilitate the maturation, migration, and activation of a variety of immune cells, including DCs, T cells, and natural killer cells (NKs), thereby initiating innate anticancer immunity (24, 25). Overall, the cGAS-STING signaling pathway can stimulate and enhance innate and adaptive antitumor immune responses through the production of cytokines such as IFN-I.

## The role of STING agonists-based nanomedicines in activating cGAS-STING pathways within tumor microenvironments

**Figure 2:**
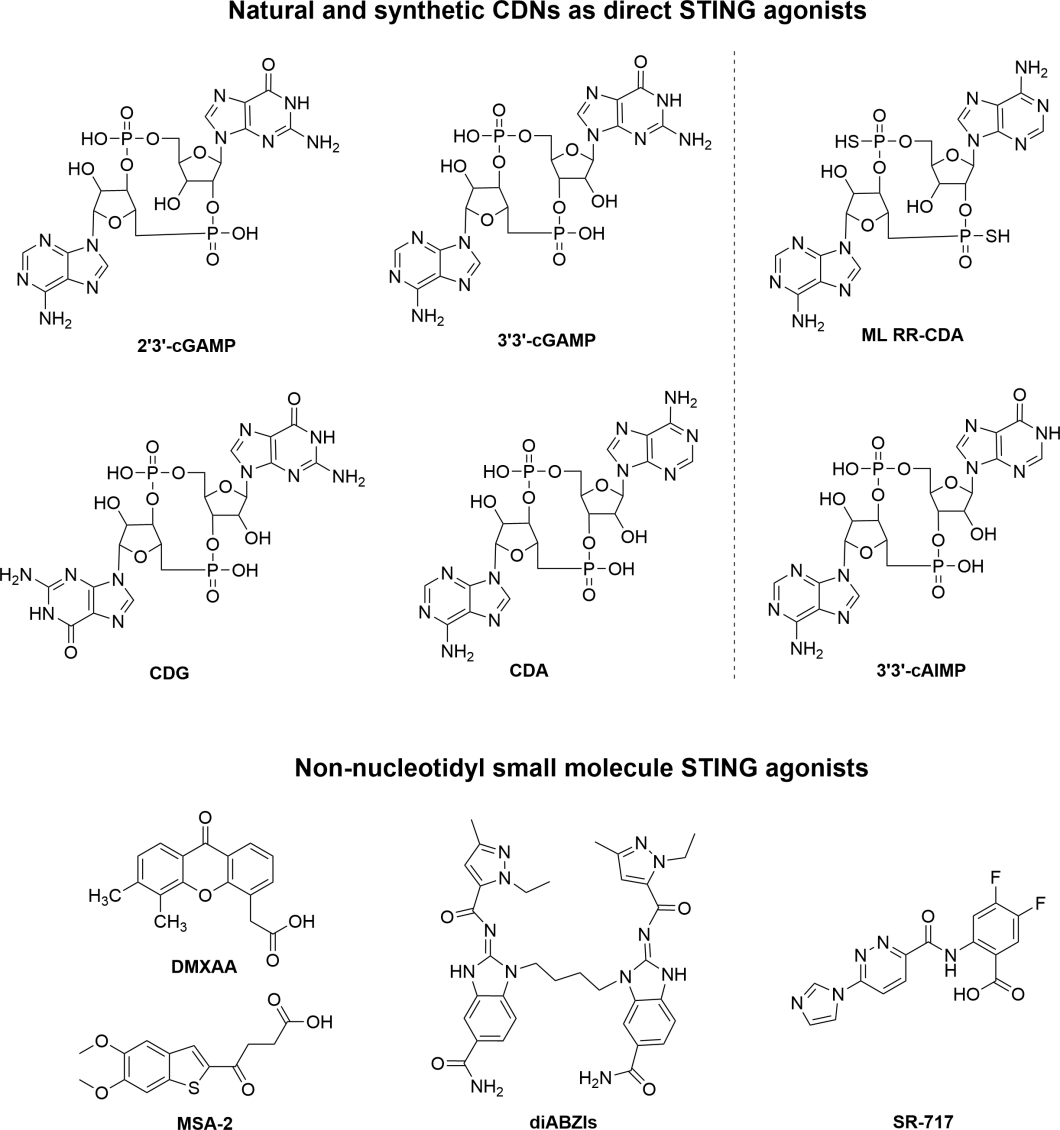
The chemical structure of representative nucleotide and non-nucleotidyl STING agonists.

In recent years, researchers have been able to demonstrate that the cGAS-STING pathway plays a crucial role in linking innate and adaptive immune responses against tumors. It would therefore be feasible to treat cancer by pharmacologically activating the cGAS-STING pathway. Natural or synthetic CDNs such as 2’3’-cGAMP, ADU-S100 were first identified as direct STING agonists. Furthermore, small molecules that are non-nucleotidyl agonists of STING were found, such as 5,6-dimethylxanthenone-4-acetic acid (DMXAA), dimeric amidobenzimidazoles (diABZI), MSA-2 and SR-717 (26) (**Figure 2**).

Therapeutic efficacy of STING agonists is severely limited by their inability to target and maintain intracellular stability. A number of small molecule STING agonists do not possess a favorable pharmacokinetic profile. In the case of exogenously administered CDNs, for example, due to their anionic nature and high water solubility, they suffer from poor cellular targeting, rapid clearance, and low transport efficiency across the cell membranes, resulting in only a very limited therapeutic effect (27, 28). To achieve satisfactory therapeutic effects, repeated local or systemic administration is required. Furthermore, as STING protein is widely expressed in normal tissues, STING agonists will inevitably cause abnormal activation of the innate immune system following large-scale local or systemic administration, resulting in autoimmune disease (29). It remains an urgent challenge to improve the delivery efficiency of STING agonists while reducing the adverse effects associated with systemic immune activation.

As a result of advances in nanomedicine, STING agonists can now be delivered more efficiently to the target cells by passive or active targeting. In comparison with small molecule STING agonists, nanoparticle-based STING agonists can prolong blood circulation, enhance drug release, and prevent degradation in lysosomes (30). As nanoparticles have enhanced permeability and retention (EPR) effect, drugs loaded on them can enhance tissue targeting without altering their original molecular structure, allowing for efficient delivery to the cytoplasm. In the past decade, it has been shown that nanomedicines like liposomes, polymeric particles, and gels can improve the bioavailability of many drugs (31). Using gel nanodrugs, for example, the drug can be released over a longer period of time and be maintained at a high level at the site of the lesion for much longer periods of time (32). Due to these unique advantages, many STING agonists are being designed as nanomedicines (33).

## Nanomedicine targeting activation of STING in the cancer immune microenvironment

In recent years, nanomedicines for activating STING have been extensively investigated for their ability to exhibit better antitumor effects. As part of this paper, we will review nanomedicine drugs loaded with different STING agonists.

### Nanomedicine loaded with natural or synthetic CDNs as STING agonists

Several natural CDNs have been found to target STING proteins, including 2’3’-cGAMP, 3’3’-cGAMP, cyclic dipolyguanosine phosphate (CDG), and cyclic dipolyadenosine phosphate (CDA). Among them, 2’3’-cGAMP, as a second messenger of information transmission in mammals, is a strong-affinity STING stimulator. The unique 2’-5’ bond in 2’3’-cGAMP makes it the most dynamic hSTING activator of all CDNs. First discovered in bacteria, 3’3’-cGAMP, CDG, and CDA molecules are analogs of 2’3’-cGAMP molecules, and they all have a certain affinity for STING proteins (34). However, it should be noted that all existing natural CDNs cannot be able to directly enter target cells due to their highly hydrophilic structures. Furthermore, CDNs are readily hydrolyzed and inactivated by phosphodiesterases, which are ubiquitous in the body. There is no doubt that loading CDN onto nanomedicine will have a more effective antitumor effect than using CDN alone.

**Figure 3:**
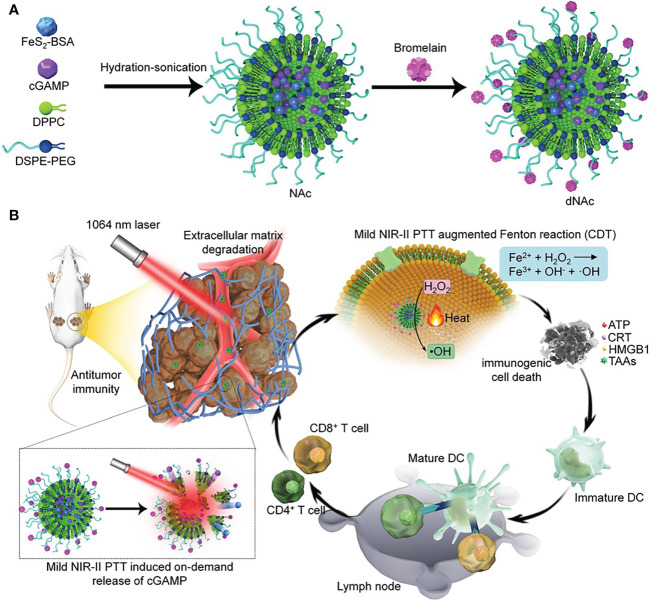
Nanomedicine loaded with 2’3’-cGAMP as STING agonists for tumor immunotherapy. **(A)** Schematic illustration of the synthesis route of ECM-degrading LNPs (dNAc). **(B)** Schematic illustration of NIR-II photoactivation of dNAc for mild photothermal effect-augmented CDT combined with STING activation for immunotherapy. Reproduced from Ref. (36) with permission. Copyright 2022 Springer Nature.

There is a growing interest in the use of lipid nanoparticles (LNPs), including liposomes, solid lipid nanoparticles (SLNs), and positively charged lipid-nucleic acid complexes (lipoplexes), to deliver STING agonists (35). For example, Lu’s group reported an ECM-degrading LNPs (dNAc) encapsulated with 2’3’-cGAMP and ferrous sulfide (FeS_2_) for long-range on-demand release of agonists to activate the STING pathway under NIR-II photoirradiation, thus constructing mild photothermal-enhanced chemodynamic-immunotherapy (**Figure 3**) (36). In this study, FeS_2_ was used as a NIR-II photothermal sensitizer as well as a Fenton catalyst. When exposed to NIR-II photoirradiation discontinuously, the nanoagonist produced mild heat that not only enhanced the potency of the Fenton reaction to lyse cancer cells, but also increased the release of 2’3’-cGAMP. Under the premise of ECM degradation, the activation of the STING pathway synergized with immunogenic cell death (ICD) enhances antitumor immune function, thereby promoting the infiltration of effector T cells into tumor tissues. As a result, it is achieved that restraint of both primary tumors and distant ones, as well as suppression of liver and lung metastases.

**Figure 4:**
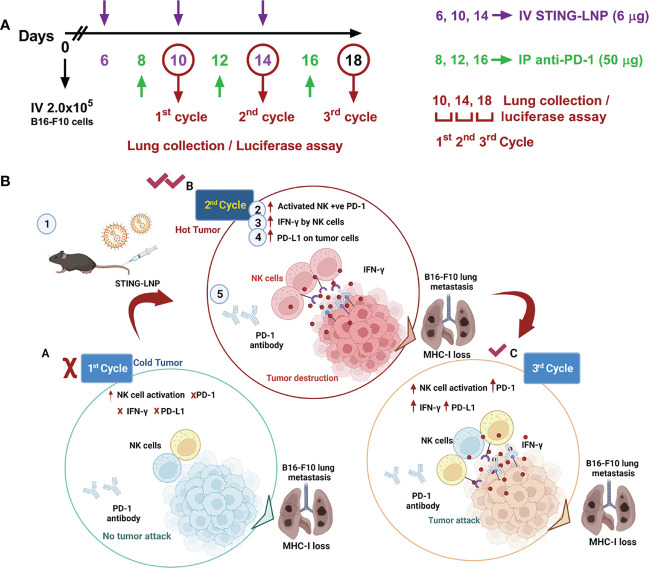
Interval- and cycle-dependent combination of CDG-loaded lipid nanoparticles with PD-1 antibody for tumor immunotherapy. **(A)** Treatment schedule for assessing the impact of dosing interval and cycle number. Cycles were counted from mice intravenously injected with B16-F10-Luc2 cells. PBS or the CDG-loaded STING-LNP were intravenously injected while the PD-1 antibody was injected intraperitoneally. **(B)** Tumor internal environment transformation and underlying mechanism behind the cycle-dependent combined activation of STING-LNP and PD-1 antibody in a lung metastasis model. Reproduced from Ref. (41) with permission. Copyright 2022 ELSEVIER.

As a bacterial second messenger, the receptor for CDG is DDX14 in the cytoplasm. It can form a complex with STING protein to activate the downstream TBK1-IRF3 pathway, which in turn induces an increase in IFN-I. To overcome cell membrane barriers, Harashima’s research group reported a lipid nanoparticle to encapsulate CDG to form a CDG/YSK05 liposome (37, 38). It allows smooth entry of CDG into the cytoplasm and successfully induces antitumor immunity by stimulating CD8^+^ T cells and NK cells. The experimental results showed that CDG/YSK05 liposome could enhance the expression of CD80, CD86, and MHC-I. These results suggest that CDG enters the cytoplasm under the transfer of YSK05 liposomes and plays a momentous role in activating the innate immune system. Subsequently, Harashima’s group reported a combinatorial strategy to combine CDG-loaded nanoliposomes (STING-LNP) with immune checkpoint inhibitors (ICIs), which reduced the drug resistance of ICIs and exerted a synergistic effect on antitumor (39). Different from CDG/YSK05 liposome, the upgraded STING-LNP uses YSK12-C4 as the main liposome material (**Figure 4**). The results suggested that stimulation of the STING pathway by SING-LNP led to the activation of PD-1-expressing NK cells, leading to the secretion of IFN-γ, which in turn upregulated the expression of PD-L1 in tumor cells. It explains for the first time why activation of STING can enhance the expression of PD-L1 in tumor cells. This conclusion is consistent with the claim that the antitumor activity of tumor immunotherapy can be assessed by the immune status in the TME (40). Furthermore, their research group also paid close attention to the importance of the treatment protocol setting of the combined application of SING-LNP and PD-1 antibody, such as the number of cycles of administration, and the order and interval between therapy administration (41).

Compared to natural CDNs, the sulfur-substituted analog ML RR-CDA (ADU-S100) stands out for its stability, lipophilicity, and high efficiency. As a result, it is considered as the most promising CDN encapsulated in nanoparticles. For instance, Doshi and coworkers recently developed a targeted cationic liposomal nanoparticle (SA-TL) loaded with ADU-S100 (STING agonist, SA) for systemic intravenous administration (42). By modifying with the WH peptide on the surface, the SA-TL can target the specific receptor Clec9a on the surface of *Batf*3 (CD103^+^) DCs, thereby mediating the entry of liposomes into CD103^+^ DCs and activating the STING pathway. Importantly, CD103^+^ DCs have been shown to be major producers of CXCL9 and CXCL10, which are IFN-I-induced chemokines that recruit T cells in a STING-dependent manner. As a result, SA-TL can accurately induce the infiltration of T cells in the tumor site by targeting CD103^+^DC, and achieve remodeling of the TME and potent antitumor immunotherapy. In this study, SA-TL exhibited robust immune responses and significant antitumor effects, whether used as monotherapy or in combination with anti-PD-L1.

In contrast to conventional nanoparticles, Lin et al. identified a tumor-targeting nanoscale coordination polymer (ZnCDA) loaded with CDA and zinc (Zn) (43). According to *in vivo* studies, intravenously administered ZnCDA prolongs CDA half-life and facilitates tumor targeting, providing an important pharmacokinetic advantage. At a single dose, antitumor effects were observed in a variety of preclinical tumor models. Notably, this study demonstrated that ZnCDA enhanced tumor accumulation by destroying endothelial cells in the tumor vasculature, revealing a novel mechanism of ZnCDA tumor accumulation. Moreover, ZnCDA was found to preferentially target and regulate antigen processing and presentation by tumor-associated macrophages (TAM), thereby further promoting antitumor T cells responses. In conclusion, ZnCDA enhances tumor targeting of CDA, and modulates TAM to improve TME. Additionally, it can be combined with ICB or IR to overcome tumor resistance.

To overcome the CDNs-related delivery barriers, many nanoplatforms have been developed to achieve effective delivery of CDNs. **Table 1** lists the effective delivery of different types of CDNs under different nanoplatforms.

**Table 1:** Recent researches on nano-delivery of CDNs for tumor immunotherapy.

Activator	Formulation	Nanoplatforms	Tumor models	ROA	Refs
**3’3’-cGAMP**	*Liposomes	Soy-PC-DOTAP liposome	Basal-like TNBC of C3 (1) Tag model and C3 (1) Tag GEM; B16-F10 (melanoma)	i.v.	(44)
**2’3’-cGAMP**	Polymers	PEG-DBP copolymers	Neuroblastoma	i.t.	(45)
**2’3’-cGAMP** **and tumor antigens**	Polymers	PEG-DBP copolymers	B16-F10 (melanoma)	i.v.; i.t.	(27) (46)
**2’3’-cGAMP** **and tumor antigens**	Polymers	pH-responsive multivesicular polymeric NPs	MC38 (colorectal cancer); TC-1 (cervical cancer)	i.t.; s.c.	(47)
**2’3’-cGAMP** **and tumor antigens**	*Polymers	the peptide-expressed biomimetic cancer cell membrane (EPBM)-coated nanovaccine (PLGA/STING@EPBM)	B16-OVA (melanoma); 4T1 (breast cancer)	s.c.; i.p.	(48)
**2’3’-cGAMP** **and FeS_2_**	Liposomes	A thermal-responsive liposome (dNAc)	4T1 (breast cancer)	i.v.	(36)
**2’3’-cGAMP**	Liposomes	PEGylated cationic liposomes	B16-F10 (melanoma)	i.t.; i.v.	(49)
**2’3’-cGAMP**	Hydrogels	HA hydrogel scaffold	4T1 (breast cancer)	i.v.; i.t.	(50)
**CDG**	Liposomes	YSK05 (pH-sensitive cationic lipid with high fusogenicity)	E.G7-OVA (T cell lymphoma)	s.c.	(37)
**CDG**	Liposomes	YSK05 (pH-sensitive cationic lipid with high fusogenicity)	B16-F10 (melanoma)	i.v.	(38)
**CDG**	*Liposomes	YSK12-C4	B16-F10-Luc2 (lung metastasis)	i.v.; i.p.	(39) (41)
**CDG** **and tumor antigens**	*Liposomes	PEGylated lipid nanoparticles	EG.7-OVA (T cell lymphoma); B16-F10 (melanoma)	s.c.	(29)
**CDG**	inorganic NPs	Cationic silica nanoparticles (CSiNPs)	B16-F10 (melanoma)	i.t.	(51)
**CDA** **and Zn**	*Polymers	DOPA, 2:1:1 mixture of DOPC, cholesterol, and DSPE-PEG2000	MC38 (adenocarcinoma; liver metastasis); B16-F10 (melanoma); BL3750 (B-cell lymphoma); LLC (Lewis lung cancer); TRAMP (mouse prostate); Panc02-SIY (pancreatic cancer)	i.v.	(43)
**CDA;** **RR-CDA**	Hydrogels	Matrigel	squamous cell cancer; HPV-transformed mouse cell	i.t.	(52)
**ADU-S100**	*Polymers	Poly (beta-amino ester) (PBAE)	B16-F10 (melanoma)	i.t.	(53)
**ADU-S100**	Polymers	Chimeric polymersomes (CPs)	B16-F10 (melanoma)	i.t.	(54)
**ADU-S100**	*Liposomes	CD103^+^ DC targeted cationic liposomal nanoparticle	MC38 (colorectal cancer); B16-F10 (melanoma)	i.v.	(42)
**ADU-S100**	Hydrogels	STINGel	MOC2-E6E7 (Oral cancer)	i.t.	(55)

*It indicats that the nanoformulation is used in combination with immune checkpoint inhibitors in the study.

ROA, routes of administration; i.v., intravenous injection; i.t., intratumoral injection; s.c., subcutaneous injection; i.p., intraperitoneal injection; Soy-PC-DOTAP liposome, hydrogenated (soy) L-α-phosphatidylcholine (Soy-PC) -1, 2-dioleoyl-3-trimethyl-ammonium-propane (DOTAP) liposome; PC7A, an ultra-pH sensitive polymer with a cyclic tertiary amine of the 7-membered ring; TNBC, triple-negative breast cancer; Resiquimod (R848), a toll-like receptor agonist; TAA, tumor-associated antigen; PEG-DBP, Poly [(ethylene glycol)-block-[(2-diethyl aminoethyl methacrylate)-co-(butyl methacrylate)-co-(pyridyl disulfide ethyl methacrylate)]]; HA, hyaluronic acid; DOPA, 1,2-dioleoyl-sn-glycerol-3-phosphate; DOPC, 1,2-dioleyl-sn-glycerol-3-phosphocholine; DSPE-PEG2000, 1,2-diastearoyl-sn-glycerol-3-phosphoethanolamine-N-[amino(polyethylene glycol)2000].

### Nanomedicine loaded with non-nucleotidyl small molecule STING agonists

Despite the fact that CDNs in a variety of molecular structures have been shown to stimulate the immune system’s ability to combat tumors in mice, their inability to easily pass through cell membranes and their lack of stability in metabolism hinder their effectiveness and potential medical use (56). Therefore, researchers have been trying to find new STING agonists with better properties. Several non-nucleotidyl STING agonists have been reported. However, most STING agonists are species-specific between human and murine STING proteins. It was reported in 2012 that DMXAA is capable of targeting activation of STING as well as inducing the expression of IFN-β in mouse macrophages (57). However, subsequent studies on the structure-function relationship between mSTING and hSTING indicated that DMXAA has a direct stimulatory effect on mSTING only, and does not appear to have any effect on hSTING (58). Further research revealed that neither DMXAA nor its analogs could bind to hSTING (59). This explains the high potency of DMXAA in preclinical mouse studies, but the poor antitumor efficacy observed in early human clinical trials. However, new agonists targeting hSTING are continually being discovered and corresponding nanomedicines are being investigated.

#### Nanomedicine loaded with diABZI as STING agonists

In a recent study, Ramanjulu and co-workers at GlaxoSmithKline discovered a series of small molecules containing the active structure of amidobenzimidazole (ABZI) with potential STING agonism through high-throughput screening (60). Among them, diABZI is the first effective non-nucleotidyl STING agonist for intravenous use with systemic antitumor activity in a mouse model (61).

In 2021, the research group led by Zhishen Ge developed a type of nanoreactor called DiABZI and glucose oxidase (GOD)-co-loaded PEG-b-P(FcMA-co-PEMA) polymersome nanoreactors (D/G@PFc) (62). These nanoreactors are about 100 nanometers in size and have pH-responsive membranes that contain ferrocene. After being injected intravenously, the D/G@PFc nanoreactors can selectively accumulate at tumor sites. The mildly acidic conditions at the tumor site can trigger a series of reactions inside the nanoreactor. Glucose and oxygen are able to diffuse into the nanoreactor’s aqueous cavities, where they are used to produce hydrogen peroxide under the catalysis of GOD. The hydrogen peroxide is then transformed into hydroxyl radicals through a Fenton reaction that is catalyzed by the ferrocene moieties. These hydroxyl radicals are highly effective at killing cancer cells, and they also release tumor-associated antigens and fragmented DNA. This helps to reverse the immunosuppressive microenvironment of the tumor and activates the STING pathway. Additionally, the DiABZI component of the nanoreactor can be released in response to the tumor’s acidity. After being taken up by dendritic cells and interacting with STING proteins in the cells’ cytoplasm, the STING pathway is activated and promotes the maturation of the dendritic cells. These mature dendritic cells are then able to migrate to lymph nodes, where they stimulate the polarization of CD8+ T cells. The populations of CD8+ T cells in both the lymph nodes and the primary tumors are increased as a result. Overall, the D/G@PFc nanoreactors are able to suppress both primary and metastatic tumors through a combination of chemodynamic therapy (CDT), the reversal of the immunosuppressive tumor microenvironment, and the activation of the STING pathway. This represents a novel approach to cancer treatment.

#### Nanomedicine loaded with SR-717 as STING agonists

Based on high-throughput screening, Lairson et al. eventually identified a non-nucleotidyl analog of the small molecule STING agonist SR-717, which exhibited higher stability under physiological conditions compared to CDNs (63). The co-crystallization complex of SR-717 and STING protein showed that SR-717, as an analog of cGAMP, could induce a “closed” activation conformation of STING. In a mouse tumor model, SR-717 administered intraperitoneally showed significant antitumor activity and promoted the activation of immune cells such as CD8^+^ T cells, NK cells and DCs as well as antigen cross-presentation.

**Figure 5:**
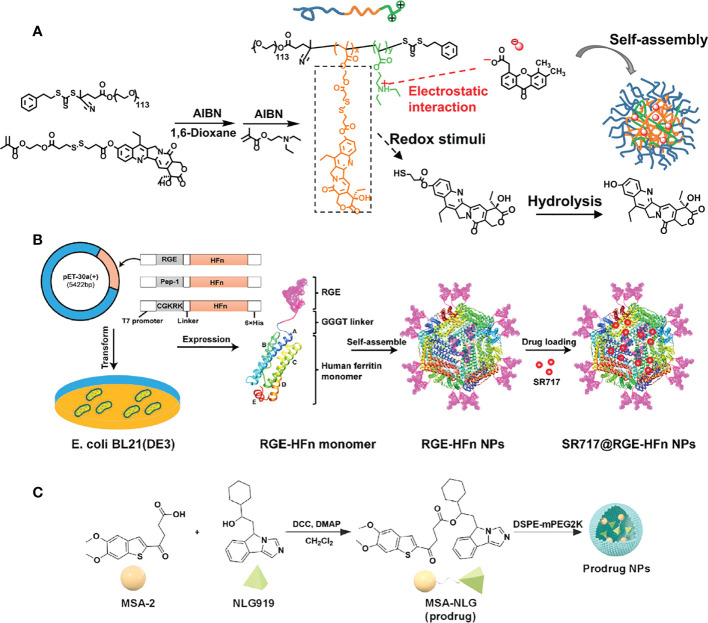
Nanomedicine loaded with non-nucleotidyl STING agonists for tumor immunotherapy. **(A)** Schematic illustration of the polymer nanoreactor (D/G@PFC) co-loaded with DiABZI and GOD via self-assembly in aqueous solution at pH 7.4. Nanopolymer membranes are responsive to the acidic pH of tumors. Reproduced from Ref. (62) with permission. Copyright 2021 John Wiley & Sons. **(B)** Schematic illustration of SR 717@RGE-HFn NP with dual targeting strategy to achieve an effective anti-glioma immune response. Reproduced from Ref. (64) with permission. Copyright 2022 ELSEVIER. **(C)** Schematic diagram of the synthesis of prodrug (MSA-NLG) obtained by encapsulating MSA-2 with DSPE-mPEG2K. Reproduced from Ref. (66) with permission. Copyright 2022 Springer Nature.

Wang et al. developed a drug delivery system for non-nucleotide STING agonist SR-717 that can be administered intravenously and effectively targets glioma brain tumors. Specifically, The N-terminus of the human heavy-chain ferritin (HFn) protein subunit was modified with tumor-targeting polypeptide RGE the by genetic engineering to endow the HFn nanocarrier with tumor-targeting and tissue-penetrating properties. Subsequently, SR-717 was encapsulated in RGE-HFn nanocarriers by pH-mediated depolymerization-reassembly to obtain SR717@RGE-HFn NPs (64) (**Figure 5**). When delivered to mice with glioma tumors, the SR-717 accumulated in the TME, leading to an immune response characterized by increased expression of STING signaling-related proteins, higher levels of proinflammatory cytokines, and increased recruitment of immune cells into the tumor tissue. As a result, the glioma growth was inhibited and the survival of the mice with glioma was improved, without causing any negative effects on blood biochemical indicators or organ pathology. This drug delivery system, which has a dual-targeting mechanism for deep penetration into the tumor tissue and effective homing of STING agonists to the glioma, provides a promising approach for the immunotherapy of glioma.

#### Nanomedicine loaded with MSA-2 as STING agonists

In a screening of 2.4 million compounds against phenotypic cell-based assays, the work by Pan et al. led to the identification of MSA-2, a chemical agonist of STING that is non-nucleotidyl (65). MSA-2 is a particularly attractive compound for clinical translation due to its oral ability.

Recently, Syeda et al. established a TME-responsive prodrug by coupling the STING agonist MSA-2 to the indoleamine 2,3 dioxygenase (IDO) inhibitor NLG919 (66). Subsequently, the prodrug was encapsulated in the DSPE-mPEG2K polymer to obtain the nano-prodrug (MSA-NLG) with high biocompatibility. Due to the esterase sensitivity of the nano-prodrug, it can specifically target the tumor site with high expression of esterase, and simultaneously break the bond to release the two components. The resulting MSA-2 molecule triggers the STING pathway to remodel immune cell populations in the TME, while NLG919 regulates IDO-mediated immune suppression. Thus, tumor immunity can be activated *in situ* and play a low-toxicity and high-efficiency antitumor effect. In this study, effective joint strategies were explored to achieve that tumor tissue-specific targeting and local amplification of immune responses safely and effectively, while reducing the side effects of immune storms.

### Nanomedicine loaded with PC7A as polyvalent STING agonists

Nanoparticle vaccine (nanovaccine) for tumor immunotherapy is an emerging hot area that has caused extensive exploration. For instance, Gao and coworkers developed a library of ultra-pH-sensitive (UPS) NPs and screened out some NPs (such as PC7A NPs (67) and PSC7A NPs (68)), whose simple mixture with antigens not only significantly enhance antigen cytoplasmic delivery and cross-presentation, but also activate the cGAS-STING pathway to stimulate innate immune responses without adjuvants. As a result, PC7A NPs incorporated different kinds of antigens, and all of them showed excellent antitumor effects in related tumor models. As research progresses, PC7A was confirmed to multimerize STING molecules for activation through polyvalent interactions. In this study, it is discovered that PC7A NPs bind to a non-competitive STING surface site which is different from the binding pocket of CDNs and non-CDNs. The experimental results show that the Glu 296-Asp 297 sites on the α5 helix of STING protein, which is responsible for PC7A binding and triggering activation. Furthermore, their follow-up studies demonstrated that PC7A NPs provided a slower but longer-lasting STING stimulation *in vitro* and *in vivo*. Based on the two characteristics of different binding sites and different action time from traditional agonists, it indicates that the combination of PC7A and 2’3’-cGAMP can achieve a synergistic therapeutic effect (69). This provides a new combination strategy for activating the STING pathway, which is to combine STING agonists with different mechanisms.

### Nanomedicine loaded with Mn^2+^ to amplify STING activation

A study conducted by James J. Moon et al. examined the potential synergistic effects of various metal ions with STING agonists and reported that Mn^2+^ had a significant effect on enhancing the activity of STING agonists (70). This study demonstrated that Mn^2+^ self-assembled with CDNs STING agonists into nanoparticles (CDNs-Mn^2+^ particle, CMP) capable of delivering STING agonists directly to immune cells and demonstrating significant therapeutic effects in several murine tumor models. The concept of ‘metalloimmunotherapy’ was presented for the first time in this study, and the potential of nanomedicine-based cancer metalloimmunotherapy was revealed.

Dai’s group fabricated a multipurpose lanthanide-doped radiosensitizer-based metal-phenolic network (DSPM), which was employed to potentiate radio-sensitization and activate the cGAS-STING pathway simultaneously, thereafter promote DC maturation and motivate robust antitumor immunity (71). The amphiphilic PEG-polyphenol was coordinated with NaGdF_4_:ND@NaLuF_4_ (radiosensitizer) *via* metal-phenolic coordination to obtain biocompatible nanoparticles, and the remaining phenolic moieties were complexed with Mn^2+^ to form DSPM. In this DSPM, amphiphilic PEG-polyphenol provides abundant coordination to lanthanide-doped radiosensitizer and Mn^2+^ to ensure stability, while ensuring the release of them due to pH-responsive. Following intravenous administration, DSPM effectively exerted the co-delivery of Mn^2+^ and radiosensitizer within the TME, resulting in the activation of the STING pathway and enhancing the sensitivity of tumor cells to X-ray. The effect of DSPM was verified to be an efficient therapeutic nanoplatforms for optimizing cancer radiotherapy in primary, distant, and lung metastatic tumor modes. In this study, the synergy between radiosensitization and Mn^2+^-mediated immunomodulation is highlighted in tumor therapy. Based on metal-phenolic coordination, Dai’s group also explore the performance of phenolic nanoadjuvant (PIMS NPs), which was self-assembled by sonosensitizer polymer (PEG-b-IR), glutathione (GSH) inhibitor (sabutoclax), TME acidic sensitive phenolic polymer (PEG-b-Pho) and Mn^2+^ (72). Among them, PEG-b-IR can trigger the generation of reactive oxygen species (ROS) by ultrasonic excitation to perform sonodynamic therapy (SDT) while Sabutoclax reduced the GSH level in tumor cells, thereby amplifying the effect of SDT. As a connecting bridge, PEG-b-Pho ensures the binding of Mn^2+^ to the nanocore to activate the STING pathway. Furthermore, together with anti-PD-1 therapy can elicit more conspicuous antitumor effects. It embodies the concept of synergistic treatment of tumors.

## Summary and outlook

In general, we have reviewed nanomedicines containing different STING agonists. From the view of STING activators, there have been developed into the current diverse situation through continuous exploration and synthesis. Not only laterally explored and designed agonists for inducing the “closed” conformation of STING, but also longitudinally discovered DNA stimulation upstream of the pathway and agonists that bond to non-competitive STING surface sites. Although a variety of STING agonists have been discovered, there are still some disadvantages in physiological barriers and targeted distribution. Furthermore, the establishment of tumor models corresponding to human tumor histology and developmental patterns is also necessary to evaluate the effects of STING agonists and to prepare for clinical translation.

As an ideal platform, the nano-delivery of cGAS-STING agonists is still in its infancy. From the perspective of drug delivery platforms, significant progress has been made in the synthesis and application of multipurpose nano-formulation materials, which greatly improving the feasibility of STING agonists for tumor immunotherapy. Moreover, modified nanoplatforms with diverse properties and functions are required to improve drug stability and achieve targeted delivery and release based on tumor cell specificity. This is significant both for improving antitumor activity and reducing the risk of the immune storm. However, multifunctional nanoplatforms present manufacturing challenges, including the complexity of large-scale production, the quality stability of different batches, tricky storage conditions, and high production costs. These are the issues that must be paid attention to and resolved for clinical translation in the future.

From the perspective of cancer immunotherapy, a single strategy cannot eliminate the tumor, and the combination strategy is more suitable to deal with the multifaceted nature of the tumor. Growing evidence suggests that STING agonists can act synergistically with other immune agents as sensitizers, including immune checkpoint inhibitors, IDO inhibitors as well as tumor antigens. In addition to immune agents, STING agonists have been proven to be synergistic with other approaches, such as chemotherapy, radiotherapy, phototherapy, and chemodynamic therapy, to effectively clear tumor masses and induce durable antitumor immune memory. Modular nanoparticles provide a platform for the co-delivery of different classes of molecules, such as tumor antigens and immunomodulators, maximizing the effect of a single drug. Moving forward, combination therapies delivered by nanoplatforms may become mainstream in cancer treatment.

## Author contributions

SC and AP wrote the manuscript. MC performed the design of the review. MC and MZ read and revised the manuscript. All authors contributed to the article and approved the submitted version.
